# Impact of heat stress and protective clothing on healthcare workers: health, performance, and well-being in hospital settings

**DOI:** 10.1093/annweh/wxaf026

**Published:** 2025-05-31

**Authors:** Razan Wibowo, Malte Satow, Caroline Quartucci, Tobias Weinmann, Daniela Koller, Hein A M Daanen, Dennis Nowak, Stephan Bose-O´Reilly, Stefan Rakete

**Affiliations:** Institute and Clinic for Occupational, Social and Environmental Medicine, University Hospital, LMU Munich, Ziemssenstr. 5, Munich D-80336, Germany; Institute and Clinic for Occupational, Social and Environmental Medicine, University Hospital, LMU Munich, Ziemssenstr. 5, Munich D-80336, Germany; Institute and Clinic for Occupational, Social and Environmental Medicine, University Hospital, LMU Munich, Ziemssenstr. 5, Munich D-80336, Germany; Institute for Occupational Safety and Environmental Health Protection, Bavarian Health and Food Safety Authority, Pfarrstr. 3, Munich D-80538, Germany; Institute and Clinic for Occupational, Social and Environmental Medicine, University Hospital, LMU Munich, Ziemssenstr. 5, Munich D-80336, Germany; Institute for Medical Information Processing, Biometry and Epidemiology, LMU Munich, Marchioninistr. 15, Munich D-81377, Germany; Faculty of Behavioural and Movement Sciences, Vrije Universiteit Amsterdam, De Boelelaan 1105, Amsterdam 1081 HV, The Netherlands; Institute and Clinic for Occupational, Social and Environmental Medicine, University Hospital, LMU Munich, Ziemssenstr. 5, Munich D-80336, Germany; Institute and Clinic for Occupational, Social and Environmental Medicine, University Hospital, LMU Munich, Ziemssenstr. 5, Munich D-80336, Germany; Department of Public Health, Health Services Research and Health Technology Assessment, UMIT—University for Health Sciences, Medical Informatics and Technology, Eduard-Wallnöfer-Zentrum 1, Hall in Tirol 6060, Austria; Institute and Clinic for Occupational, Social and Environmental Medicine, University Hospital, LMU Munich, Ziemssenstr. 5, Munich D-80336, Germany

**Keywords:** healthcare workers, heat stress, physiological monitoring, protective clothing, well-being

## Abstract

**Introduction:**

Heat stress poses a recognized threat to human health. Despite growing evidence, its impact on healthcare workers (HCWs) remains underexplored. This study evaluates occupational heat stress in HCWs, assessing physiological responses and subjective well-being.

**Methods:**

Twelve HCWs from a German university hospital were monitored in non-air-conditioned intensive care units (ICU) and non-ICU settings during the summer of 2022 (mean indoor temperature of 26.5 °C) and again in the autumn of the same year or in March 2023 (mean indoor temperature of 23.6 °C). Physiological data (core body temperature, heart rate, and skin temperature) and subjective perceptions were measured using wearable sensors and questionnaires.

**Results:**

In summer, mean core body and skin temperatures were higher by 0.4 °C and 0.3 °C, respectively. ICU workers exhibited higher heart rates and reported greater mental demands, frustration, and discomfort, particularly when using personal protective equipment (PPE). Common symptoms included sweating, fatigue, and headaches.

**Conclusion:**

We observed some evidence suggesting that elevated indoor temperatures and reported PPE usage contribute to increased HCWs’ heat strain, which could potentially affect health, safety, and performance. Given the observed trends, we recommend considering cooling vests and revising workplace standards to mitigate heat stress.

**What’s Important About This Paper?**

This study shows that heat exposure and personal protective equipment use negatively affect healthcare workers’ perceived stress and health. The study also highlights gaps in awareness and existing measures addressing occupational heat stress in Germany, offering insights to improve strategies for reducing heat stress among healthcare workers.

## Introduction

Climate change has amplified occupational heat stress, particularly among healthcare workers (HCWs) who are exposed to increased physical demands while caring for patients and working in non-climatized environments (Pinkerton and Rom 2014; Kenny et al. 2018; Schoierer et al. 2020). This raises concerns about their health, performance, and well-being (Flouris et al. 2018; Casanueva et al. 2019; Ebi et al. 2021). Extreme weather events, including heatwaves and prolonged hot summer days (>30 °C), are increasing in frequency and intensity, exacerbating occupational risks (Papalexiou et al. 2018; Bisolli et al. 2019). In Germany, record-breaking hot summers in recent years (2003, 2015, 2018, and 2023) have exposed workers in non-air-conditioned environments to heightened risks of heat stress and related health impacts (Hoy et al. 2020; Stanley et al. 2023). Addressing this issue is particularly important given the prevalence of non-air-conditioned spaces in German clinics and hospitals (Nicolas et al. 2019). To our knowledge, no previous study has systematically assessed thermal stress and strain in healthcare facilities, including hospitals, in Germany.

Heat stress, defined as the physiological strain caused by exposure to elevated temperatures, poses significant threats to health and productivity, especially in occupational settings (Cheung et al. 2000; Cramer and Jay 2016; Cramer et al. 2022). Prolonged exposure to temperatures above 26 °C can lead to heat strain, impairing thermoregulation and potentially causing fluid loss (dehydration), resulting in fatigue, headaches, and dizziness (Sessler 2008; Périard et al. 2021). Such conditions may also reduce work capacity and productivity (Borg et al. 2021; Quartucci et al. 2024), while increasing the risks of workplace errors or injuries (Xiang et al. 2014; Borg et al. 2021). These impacts can create a cascading effect—heightening the risk of heat-related health issues and contributing to economic burdens (Borg et al. 2021). A meta-analysis by Flouris et al. (2018) estimated a 30% decrease in work productivity for both indoor and outdoor occupations during heat stress conditions, with a 2.6% decline in productivity for each degree above 24 °C Wet Bulb Globe Temperature (WBGT) (Flouris et al. 2018). The WBGT index integrates temperature, humidity, wind speed, and solar radiation to provide a more comprehensive assessment of heat stress on the human body (Mazlomi et al. 2017; Dillane and Balanay 2020).

HCWs face compounded risks due to the physical demands of patient care and the use of personal protective equipment (PPE) (Bose-O’Reilly et al. 2021; Verbeek et al. 2021). PPE, although essential for infection control, it impairs heat dissipation mechanisms, particularly on hot summer days, further amplifying physiological strain (Cramer and Jay 2016; Eggenberger et al. 2018). Discomfort associated with PPE usage has raised concerns around compliance and its impacts on performance and well-being (Jegodka et al. 2021; Chaudhari et al. 2023). Combined with the often-inadequate attention paid to the health concerns of HCWs, these challenges can lead to increased dissatisfaction in the workplace (Schoierer et al. 2020; Günster et al. 2021).

Wearable technologies provide new opportunities to monitor physiological responses in real-time, offering insights into heat stress impacts (Teng et al. 2008; Bonato 2010; Chang et al. 2022; Wibowo et al. 2023). Despite these advances, their application in healthcare settings remains limited due to concerns about interference with medical equipment (eg in x-ray rooms), sensor reliability, and worker comfort.

Given the gaps in understanding heat stress among HCWs, this study primarily aimed to address these issues by evaluating both physiological and subjective responses to heat stress, contributing to the development of improved workplace strategies and safety standards.

## Methods

### Study design and setting

This observational study employed convenience sampling to recruit HCWs from intensive care units (ICU) and non-ICU settings at the University Hospital of LMU Munich. The observational design was chosen to assess real-world conditions without altering participants’ daily routines. Participants completed two observation periods: during summer (June to August 2022) and fall (October to November 2022) or spring (March 2023). The study was conducted in accordance with the ethical standards outlined in the Declaration of Helsinki for research involving human subjects and received approval from the ethics committee at the Medical Faculty of LMU Munich (#21-0197).

### Participants

Eligibility criteria required employment as HCW at the University Hospital of LMU Munich and proficiency in written German. All interested HCWs who contacted us to participate were invited to an introductory session. During this session, comprehensive study information was provided and informed consent (including data protection consent) was obtained. Baseline health assessments (including age, weight, height, forehead temperature, and heart rate—HR) were conducted. In addition, participants completed a customized questionnaire addressing heat stress in the workplace to assess subjective heat stress perception, knowledge of existing countermeasures, and workplace conditions, such as the availability of air-conditioning systems. Upon completion of both observations, each participant was compensated with EUR 50.

### Monitoring heat stress environment

Environmental parameters were recorded using the *QUESTemp 34* Environment Thermal Monitor (Quest Technologies, Wisconsin, USA) (see Supplementary Figure S1), following established protocols to avoid interferences and ensure data accuracy (eg no barriers or objects that could radiate heat, such as electronic devices or heaters, and no person approaching the device during the measurement). This device provided the WBGT index, which is considered the “gold standard” for quantifying the effects of occupational heat stress in various studies (Dillane and Balanay 2020; Ioannou et al. 2022; Hall et al. 2023). Environmental data were recorded at 1-min intervals.

### Questionnaire assessment

Generally, the questionnaire to capture participants’ subjective perceptions of heat stress at work was administered at two time points: at the beginning of each shift (eg for the question on current health status using a 0-to-10 Visual Analogue Scale (VAS)) and at the end of each shift (for all other questions). On the VAS, it was administered before and after each observation with 0 representing the worst health and 10 representing the best health (Delgado et al. 2018). In addition, the NASA Task Load Index (NASA-TLX) was used to assess the mental and physical workload, stress levels, and well-being (Hart and Staveland 1988). The NASA-TLX consists of six dimensions (subscales) and participants were asked to rate each dimension on a scale of 0 to 10, with 0 indicating very low workload or demands and 10 indicating very high workload or demands. For the subscale “performance” only, 0 indicates poor performance and 10 indicates high or best performance. Mean scores were calculated for each subscale.

**Fig. 1.**
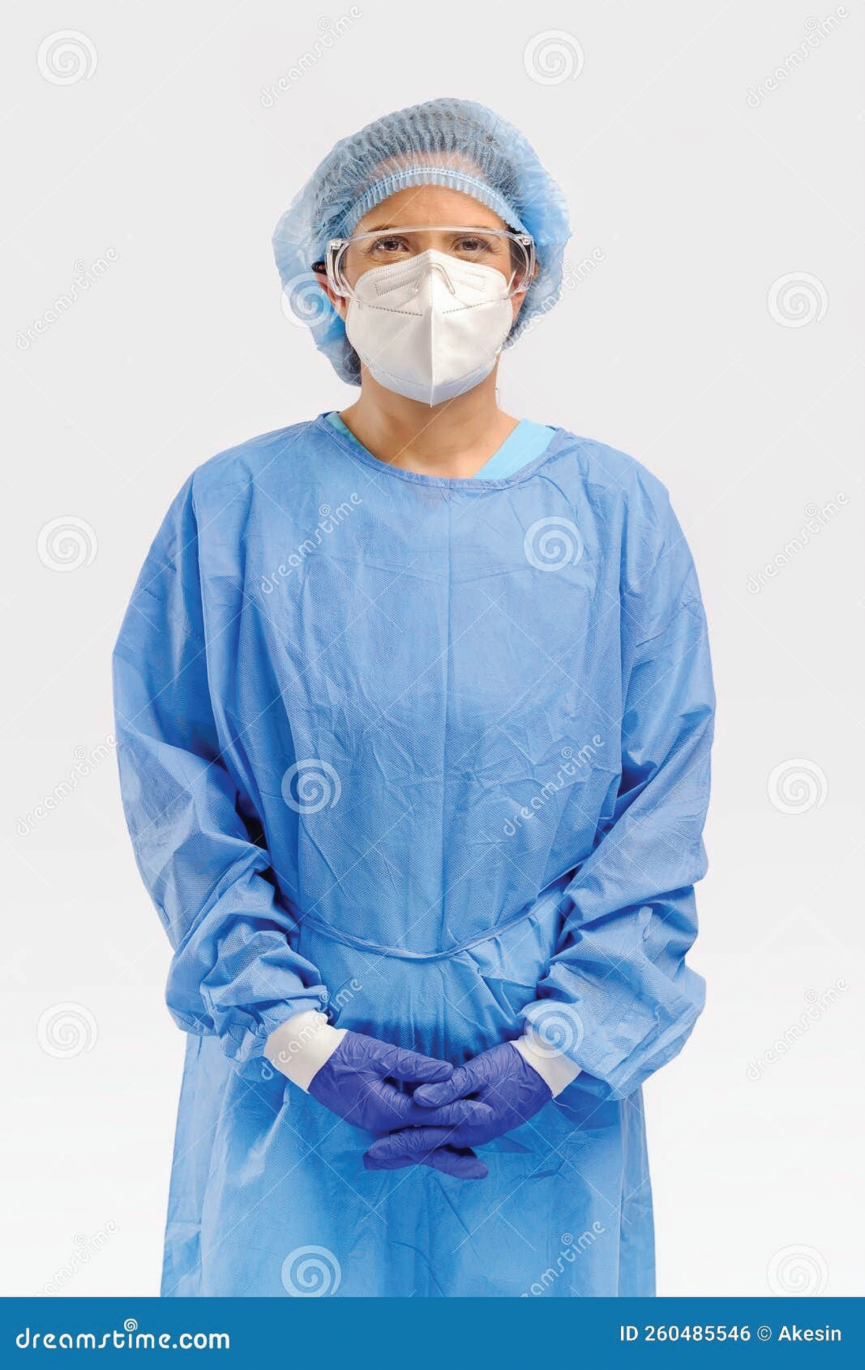
Example of PPE used by HCWs, including a protective isolation gown, disposable purple nitrile gloves, safety glasses, a white FFP2/N95 mask, and a surgical bonnet. The PPE is essential for infection prevention in hospital settings. *(Image source: Dreamstime, ID 260485546, © Akesin*)

In addition, we assessed heat stress in HCW using questions similar to those used by Jegodka et al. (2021). These included a 0 to 10 scale for perceived physical, psychological and thermal stress at work, a 4-Likert scale (“no“, “rather no,” “rather yes,” and “yes”) for typical physical discomfort experienced during nursing work, the amount of water/liquid consumed, and the intensity of HCW-related activities performed, including the use and duration of use of specific PPE such as protective gowns, disposable gloves, safety glasses, surgical/FFP2/N95 face masks, and surgical bonnets (see Fig. 1).

### Monitoring of physiological parameters

Physiological responses were monitored throughout the observation period, which lasted for half of a standard work shift (or approximately 3.5 to 4 h). Data were recorded at consistent times during both observation periods (eg the early shift in observation 1 was also applied in observation 2) to ensure consistency. Validated wearable devices were used for continuous monitoring: (i) the c*osinuss° Two* (Cosinuss GmbH, Munich, Germany) in-ear sensor (Supplementary Fig. S2A) (Ellebrecht et al. 2022) and (ii) the *Thermochron iButton* (CK electronic GmbH, Cologne, Germany) temperature loggers (Supplementary Fig. S2B) (van Marken Lichtenbelt et al. 2006).

While parameters such as body temperature (BT) and HR were monitored using the in-ear sensor, participants’ skin surface temperatures were recorded using the button loggers at multiple body sites (left/right infraclavicular, abdomen, and left/right midthigh). The minimum number of skin sites required to accurately estimate mean skin temperature in the heat is four, as temperature variations across different body locations remain minimal in such environments (ISO_9886 2004). The average of these five sensor locations was taken as the mean skin temperature. These body sites were selected to be under the PPE to minimize the influence of external environmental factors and avoid interference with HCWs’ activities during their shifts. Prior to observation, each participant selected the appropriate in-ear sensor size (small or medium). A small belt pouch containing the sensor receiver was worn during the observations. Given concerns about ear sensor coverage (± 5 m), this step was necessary to ensure uninterrupted recording and transmission of data. Data were recorded at 1-s intervals for the in-ear sensor and at one-minute intervals for the skin temperature loggers.

### Data handling and statistical analysis

Environmental and physiological data were measured outside the observation period, and data with apparent sensor malfunctions were removed. In our in-ear sensor, the quality of the HR measurement was determined by the quality index, which is an algorithm that quantitatively assesses the functional near-infrared spectroscopy signal quality on a numerical scale from 0 (very low quality) to 100 (very high quality) (Ellebrecht et al. 2022). Only HR data with a corresponding measurement quality index of 50 or higher were included. For BT, the remaining data were used for the analysis. In addition, for the analysis of peripheral temperature data, the measured skin temperature data were corrected using an external calibration, and the mean skin temperature (MST) was calculated for one observation of each participant as the average of the five skin logger sites.

Descriptive statistics summarized key variables. Five-minute means were calculated for BT and HR to match the interval of the skin sensors. All variables were tested for normal distribution. For non-normally distributed parameters, boxplots with non-parametric tests (ie Wilcoxon rank-sum) were utilized. Normally distributed parameters were assessed using parametric tests (ie *t*-tests). Given the complexity and diversity of our physiological data, simple parametric tests, such as *t*-test and/or ANOVA, may lack the depth to capture nuances and trends in the time series, as previously discussed in our publication (Wibowo et al. 2023). Therefore, we used a mixed-effects model to account for longitudinal variation.

A separate mixed linear model was fitted for each physiological outcome. The fixed effects in these models included observation time, ambient temperature (warm/normal), and ward type (ICU/non-ICU) as the main predictors. The random effects included random intercepts and random slopes, allowing the models to capture the variation between individuals and changes over time. Although the distribution of potential confounders such as BMI, age, and sex may be uneven, these factors are important and were controlled for in the analysis. The alpha (α) level was set at 0.05 for all statistical tests. All *P*-values were two-tailed. Data cleaning process and statistical analysis were performed using R statistical software version 4.1.3.

## Results

### Participants’ characteristics and heat stress-related topics

Of the 19 initial participants, 12 HCWs were completed in both observation periods (ICU: *n* = 6; non-ICU: *n* = 6). Most participants were female (*n* = 10) and had over 5 years of professional experience. Their mean age was 33.7 ± 9.7 years old (range: 24 to 51). No significant differences were observed between ICU and non-ICU participants in age, height, or weight, although forehead temperature and blood pressure showed statistically significant differences (Table 1).

**Table 1.** Baseline characteristics of participants.

Characteristic	ICU (*n* = 6) Mean ± SD	Non-ICU (*n* = 6) Mean ± SD	*P-*value^a^
Age, years	32.2 ± 8.4	35.2 ± 9.9	0.12
Forehead temperature, °C	36.6 ± 0.5	36.4 ± 0.3	0.005**
Height, m	1.7 ± 0.0	1.7 ± 0.1	0.84
Weight, kg	66.3 ± 14.4	69.7 ± 11.3	0.07
Blood pressure (sys/dia), mmHg	119/76 (± 8.3/8.9)	128/85 (± 16.9/9.3)	0.03*
Resting heart rate (beats/min)	69 ± 4.7	69 ± 9.8	0.23

^a^As all variables are quantitative, *t*-test was employed to test the significance of difference.

Significance levels: ***P < 0.001, **P < 0.01, *P < 0.05.

### Environmental characteristics

During summer, the average indoor dry bulb temperature was 26.5 °C (±1.5 °C), with a corresponding WBGT of 21.9 °C (±1.3 °C). In fall/spring, these values were lower: 23.6 °C (±1.8 °C) and 19.5 °C (±1.3 °C), respectively. These measurements indicate heightened temperatures for indoor working environments during the summer.

### Subjective perceptions of stress and workload

Considering participants’ self-reported health status, pre-post differences were calculated for each observation. On average, a slightly higher negative difference was observed during the warm summer observation period (−0.8 vs. −0.7; n.s.) (see Supplementary Fig. S3).

**Fig. 2.**
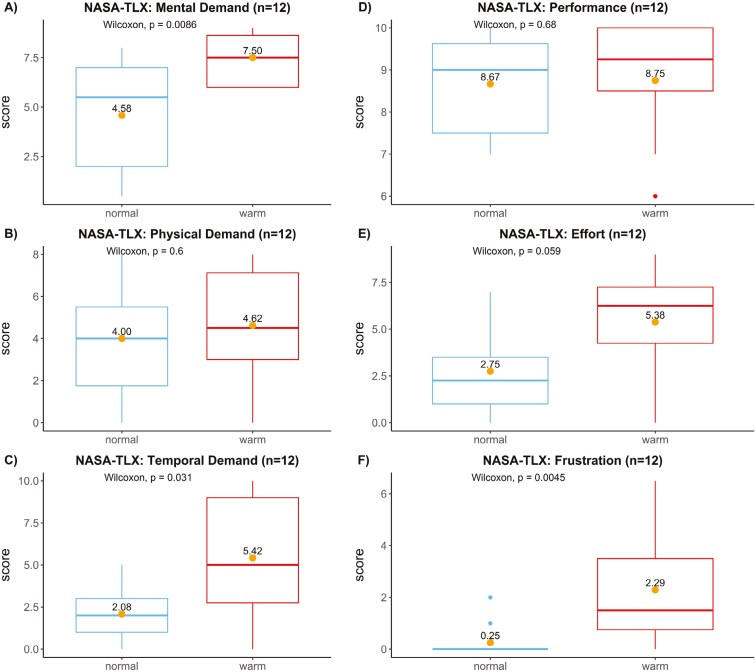
NASA-TLX ratings of stress and workload (on a scale of 0 to 10): A) mental, B) physical, and C) temporal demand, as well as D) performance, E) effort, and F) frustration. Data were presented as boxplots; the dots are mean scores and are analyzed by the Wilcoxon rank-sum test.

Participants were asked to report their mental and physical workload, stress, and well-being while performing their work (see Fig. 2). Statistically significant increases in mental (7.5 vs. 4.6; *P* = 0.01) and temporal demands (5.4 vs. 2.1, *P* = 0.03) were reported in warm compared to normal temperature. On the other hand, physical demands (4.6 vs. 4.0) and effort scores (5.4 vs. 2.8) were higher under warm environment temperatures, although not statistically significant. Moreover, participants reported significantly higher frustration scores under warm temperatures, (2.3 vs. 0.3; *P* = 0.01), suggesting increased strain.

**Fig. 3.**
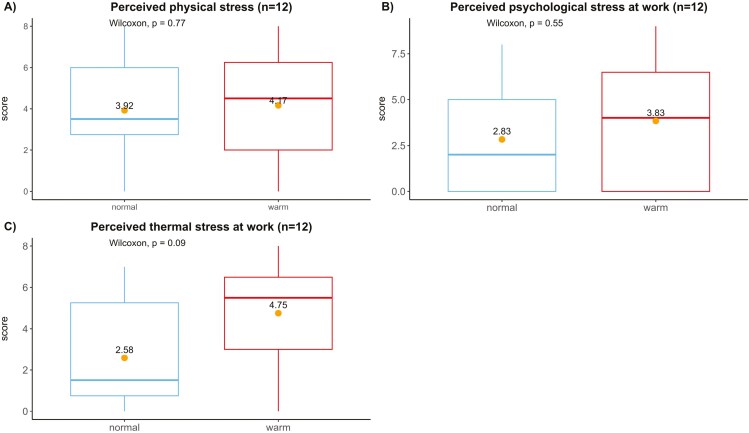
Participants’ A) physical; (B) psychological; and (C) thermal stress. All dimensions are on a 0 to 10 scale and presented as boxplots. The dots are mean scores, and these were analyzed by the Wilcoxon rank-sum test for the significance of differences.

Mean scores for physical (4.2 vs. 3.9), psychological (3.8 vs. 2.8), and thermal stress (4.8 vs. 2.6) were also calculated for the observation under warm and normal temperatures, respectively (Fig. 3).

To evaluate potential physical discomforts associated with the use of PPE, the participants reported the use and the duration of use of specific PPE during the observation period. Unfortunately, there is no information available from procurers on the manufacturer or material of these PPE. Overall, ICU participants reported more frequent and prolonged use of PPE compared to their non-ICU counterparts. Disposable gloves and protective gowns were the most frequently used PPE items in ICU environments, whereas FFP2 face masks were predominantly utilized by HCWs in non-ICU settings.

To further explore the relationship between PPE use and physical discomfort, participants were asked to report their physical symptoms associated with PPE wear (see Supplementary Fig. S4). Across all observation periods, fatigue was the most prevalent reported symptom (46%), followed by increased sweating (25%), exhaustion (25%), and headaches (17%). In addition, participants indicated that specific PPE components contributed to their discomfort. For example, prolonged use of the FFP2 face mask was commonly linked to breathing difficulties and headaches, while protective gowns were often associated with excessive sweating. These findings align with the complaints raised during the introductory session, where participants emphasized workplace burdens caused by unfavorable temperature and humidity levels, particularly in summer, exacerbating physical complaints such as breathing difficulties, excessive sweating, and dizziness. To mitigate these effects, participants implemented various personal countermeasures, such as increasing water intake and taking frequent rest periods (see Supplementary Table S1 for further details).

### Physiological parameters

**Fig. 4.**
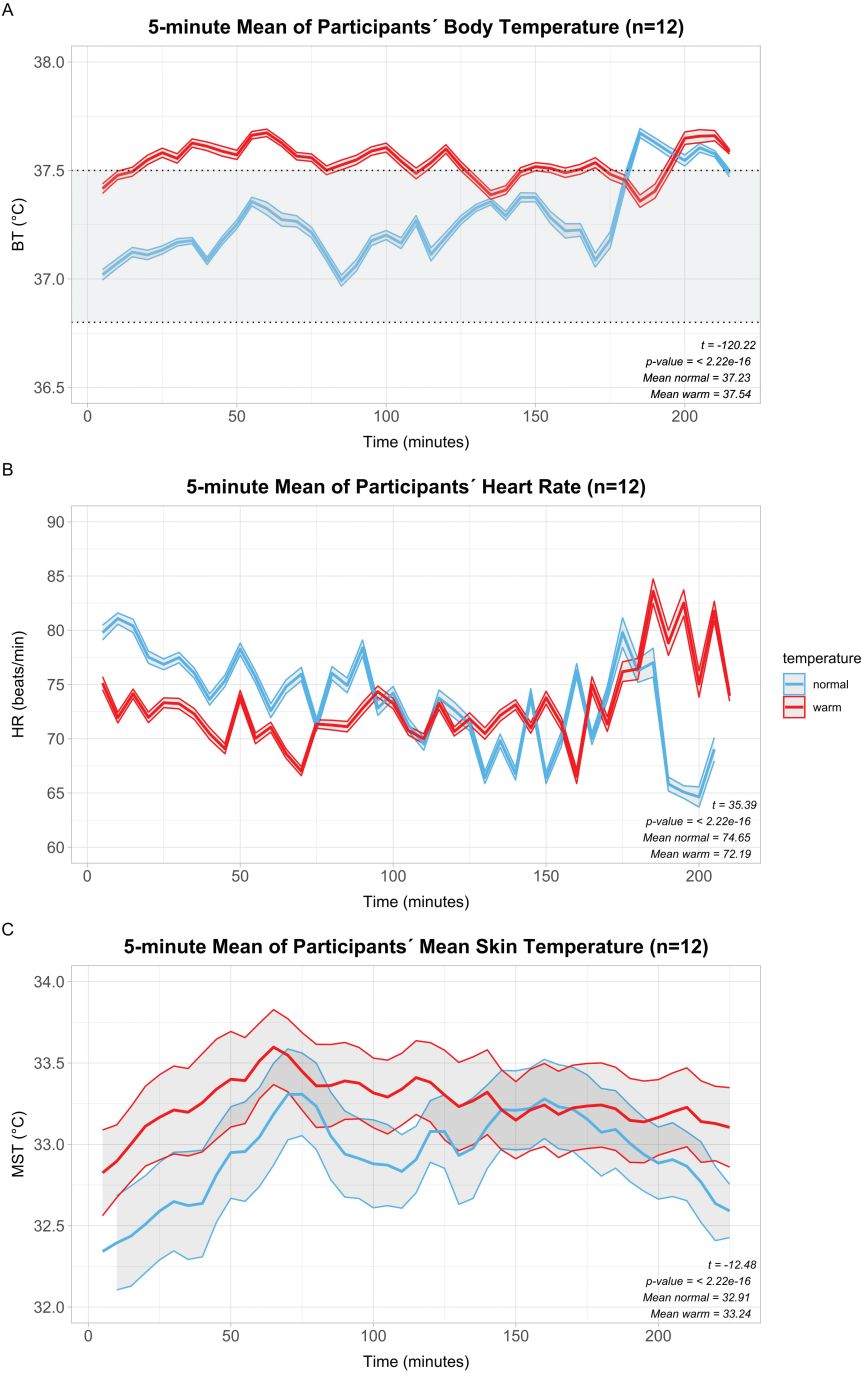
A) Body temperature; B) heart rate, both measured using cosinuss° Two in-ear sensor; and C) mean skin temperature, measured with *Thermochron iButton* plotted against time for warm (summer; red) and normal (fall/spring; blue). Data are presented as 5-min moving averages. Welch’s *t*-test was used to assess differences between seasonal conditions.

Monitoring of physiological parameters included participants’ BT, HR, and MST (see Fig. 4). Irrespective of the wards in which participants worked during the observations, participants exhibited higher average BT during summer (37.5 °C vs. 37.2 °C; +0.4 °C), with 69.8% exceeding normal temperature ranges (>37.5 °C) (Mazlomi et al. 2017; Ebi et al. 2021) (see shaded grey area in Fig. 4A). In contrast, the normal ambient temperature observed in fall/spring contributed to approximately 13.9% of BT exceeding this range.

Furthermore, the mean HR was slightly lower in summer (72 beats/minute vs. 75 beats/minute), likely reflecting thermoregulatory adjustments. Finally, the participants’ MST was higher during the warm period (33.2 °C vs. 32.9 °C; +0.3 °C). T-tests showed a statistically significant difference (*P* < 0.001) for all physiological parameters measured.

A linear mixed-effects regression analysis was performed to examine the factors influencing each physiological parameter while accounting for individual variations over time. The analysis revealed that observation time did not exert a statistically significant effect in any of the three models, indicating that time was not a substantial predictor of physiological parameters.

When compared to the fall/spring period, characterized by normal ambient temperatures, participants exhibited a statistically significant increase of approximately 0.4 °C in BT during the warm summer period. In addition, although not statistically significant, participants experienced a decrease of about 4 beats/minute in HR and an increase of 0.3 °C in MST under warm conditions.

Regarding the ward type, participants working in non-ICU wards tended to have lower values across all physiological parameters compared to their ICU counterparts. However, these differences were not statistically significant, with the exception of HR. Table 2 provides a summary of the regression coefficients for both fixed and random effects.

**Table 2.** Prediction of physiological parameters using a linear mixed-effects model (*n* = 12). Factors such as observation time, ambient temperature, and ward types were included as fixed effects in the regression model, while individual responses to these factors were considered as random effects.

	Body temperature (°C)^a^	Heart rate (beats/min)^a^	Mean skin temperature (°C)^a^
*Fixed predictors estimation (standard errors in brackets)*			
intercept	37.11*** (0.11)	85.65*** (4.77)	33.10*** (0.24)
observation time (t)	−0.00 (0.00)	−0.00 (0.00)	0.00 (0.00)
temperature (warm)	0.38*** (0.08)	−4.00 (4.53)	0.27 (0.17)
wards (non-ICU)	−0.08 (0.15)	−16.27* (6.09)	−0.48 (0.30)
*Random effects (standard errors in brackets)*			
Random intercept (ID)	0.07 (0.26)	16.20 (12.69)	0.40 (0.63)
Random slope: t (ID)	0.00 (0.00)	0.00 (0.00)	0.00 (0.00)
Random slope: temperature (warm) (ID)	0.08 (0.28)	27.41 (15.08)	0.37 (0.60)
Residual variance	0.08 (0.29)	16.67 (12.91)	0.15 (0.38)

^a^All models are adjusted for age, BMI, and sex (fixed effects) and random effects: intercept and slope. All adjusting variables are non-significant.

Significance levels: ***: *P* < 0.001, **: *P* < 0.01, *: *P* < 0.05.

## Discussion

This study examined the impact of occupational heat stress on HCWs in non-air-conditioned hospital settings. Consistent with prior research, we observed that elevated indoor temperatures were associated with higher levels of perceived physical and mental strain among participants (Lee et al. 2020; Chaudhari et al. 2023; Wibowo et al. 2023; Quartucci et al. 2024). Specifically, our results show that heat stress links to an increase in mental and temporal workload, requiring greater effort to complete routine tasks. Subjective symptoms, such as fatigue, increased sweating and headaches, reflected the combined psychological burden of heat exposure and situational PPE requirements. While some PPE were worn across both ICU and non-ICU settings, we did not find statistically significant evidence for a direct effect of PPE use on the exacerbation of heat strain in this study. Physiological measurements show elevated BT (+0.4 °C) and MST (+0.3 °C) during the warm summer months, suggesting thermal strain associated with environmental conditions.

Such findings highlight the urgent need for improved heat management strategies in healthcare settings. Workplace heat-related stress has been shown to reduce motivation, impair cognitive performance and decision-making, and compromise safety, thereby increasing the risk of occupational injuries (Xiang et al. 2014; Casanueva et al. 2019; Russo et al. 2019; Foster et al. 2020; Borg et al. 2021). Although Germany’s Workplace Regulation (German: *Arbeitsstättenregel*, ASR A3.5) specifies a 26 °C threshold for addressing workplace heat, and the Occupational Health and Safety Act (German: *Arbeitsschutzgesetz*, ArbSchG) requires risk assessments and implementation of appropriate measures (BAuA (Bundesanstalt für Arbeitsschutz und Arbeitsmedizin) 2022), awareness of these regulations among HCW remains limited. Furthermore, systematic heat protection plans—specifically tailored to healthcare facilities—have not yet been adequately developed or widely implemented (Zielo and Matzarakis 2018). Addressing this gap is critical to safeguarding the well-being and performance of HCW, particularly in relation to climate change and the increasing frequency of its subsequent environmental effects.

To our knowledge, this study represents the first German observational study quantifying the impact of heat exposure on HCW physical work capacity under real-life conditions. Unlike controlled climate chamber studies (Eggenberger et al. 2018; Wibowo et al. 2023), this investigation provides valuable insights into HCWs’ physiological and subjective responses to heat exposure in routine clinical settings. However, several limitations should be considered. First, while work intensity is a critical determinant of heat strain, our study did not systematically analyze the differential impact of specific physical activities on health and performance. To this extent, we ensured that measurements were conducted during normal daily tasks without additional metabolic load from research equipment.

Second, while we documented the types of PPE used and their predominant use across all settings, we did not systematically track the exact duration of PPE wear for each participant during their shift. Since PPE use was dictated by patient care demands, we could not mandate participants to wear or remove PPE at fixed times. This situation limits our ability to quantify PPE-specific heat effects or conclude whether prolonged PPE use influences these effects.

Third, the limited sample size and observational design may limit the generalizability of the results. Due to the real-life setting, randomization of observations was not feasible, and the long interval (~ 3 to 6 months) between two individual observations may have introduced variability in responses.

Fourth, the use of wearable devices, although practical and non-invasive for assessing heat strain, may have been subject to measurement inaccuracies. Specifically, the assessment of ear canal temperature as a proxy for core BT may have been affected by the presence of cerumen, ear canal anatomy, and ambient temperature. Nevertheless, this method has been validated as a practical and reliable tool for estimating core BT (Daanen 2006; Roossien et al. 2021). Similarly, the use of heart rate measurements via wearables is supported by existing evidence indicating their diagnostic value and utility as indicators of physiological responses to heat stress (Teng et al. 2008; Roossien et al. 2020). In addition, we recognized the importance of measuring participants’ MST as a crucial indicator of thermal sensation. While core BT remains relatively stable due to thermoregulatory mechanisms in healthy individuals, peripheral skin temperatures are highly sensitive to fluctuations in ambient temperature and metabolic activity, providing important insights into thermal responses (Krishnamurthy et al. 2017).

Another limitation pertains to the moderate average ambient temperature (26.5 °C) observed during the study, which may not fully reflect extreme heat conditions encountered during hotter summers, such as in 2023. Moreover, the findings may be less relevant in warmer regions where hospitals—or at least ICU facilities—are typically fully air-conditioned. These limitations underscore the necessity for further research, with a particular focus on extremely hot events. Subsequent studies should include larger, more diverse samples across different hospital environments, and evaluate the effectiveness of specific interventions (eg cooling vests and advanced PPE materials). In addition, research should explore the economic implications of heat stress and assess the cost-effectiveness of mitigation strategies to inform evidence-based interventions and workplace policies.

## Conclusion

This study demonstrates that elevated ambient temperatures and PPE usage exacerbate heat strain among HCWs, impairing their performance and well-being. As climate change intensifies, addressing workplace heat stress must become a priority. Implementing engineering controls, revising PPE standards, and introducing adaptive strategies can enhance occupational health and safety. Further research should focus not only on filling the gaps in science-based information but also on interventions and long-term impacts to safeguard HCWs’ health and productivity at work.

## Supplementary material

Supplementary material is available at *Annals of Work Exposures and Health* online.

wxaf026_suppl_Supplementary_Figures_S1-S4_Tables_S1

## Data Availability

The data can be made available upon reasonable request.
