# Supplementary material for: Impact of heat stress and protective clothing on healthcare workers: health, performance, and well-being in hospital settings
Source: Ann Work Expo Health. 2025 May 31;69(6):665–75. doi: 10.1093/annweh/wxaf026 (PMC12262050; doi:10.1093/annweh/wxaf026)
Supplement: wxaf026_suppl_Supplementary_Figures_S1-S4_Tables_S1 [file wxaf026_suppl_supplementary_figures_s1-s4_tables_s1.pdf]

Impact of Heat Stress and Protective Clothing on Healthcare Workers: Health, Performance and Well-being in Hospital Settings

Razan Wibowo [1], Malte Satow [1], Caroline Quartucci [1,2], Tobias Weinmann [1], Daniela Koller [3], Hein AM Daanen [4], Dennis Nowak [1], Stephan Bose-O´Reilly [1,5] and Stefan Rakete [1]

[1] Institute and Clinic for Occupational, Social and Environmental Medicine, University Hospital, LMU Munich, 80336 Munich, Germany

[2] Institute for Occupational Safety and Environmental Health Protection, Bavarian Health and Food Safety Authority, 80538 Munich, Germany

[3] Institute for Medical Information Processing, Biometry and Epidemiology, LMU Munich, 81377 Munich, Germany

[4] Faculty of Behavioural and Movement Sciences, Vrije Universiteit Amsterdam, Amsterdam, The Netherlands.

[5] Department of Public Health, Health Services Research and Health Technology Assessment, UMIT - University for Health Sciences, Medical Informatics and Technology, Hall in Tirol, Austria

Supplementary Figures

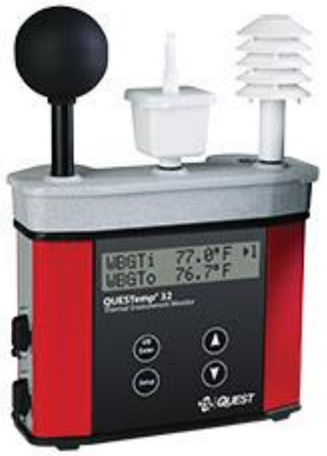

Figure S1. *QUESTemp 34*® Environment Thermal Monitor to capture heat stress environment.

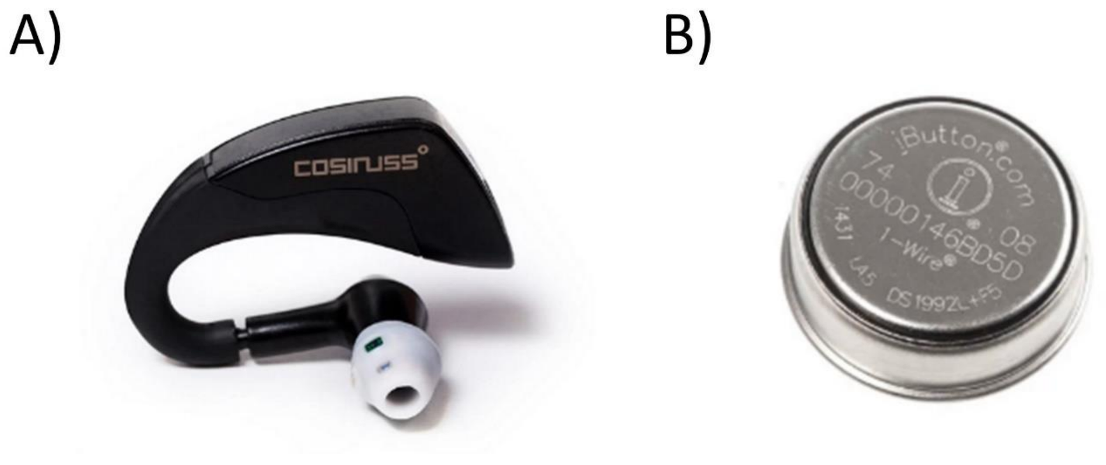

Figure S2. Wearable sensors used during the observations. *Cosinuss° Two* in-ear sensor® **(A)** to monitor body temperature and heart rate and *Thermochron iButton*® **(B)** to record skin surface temperatures.

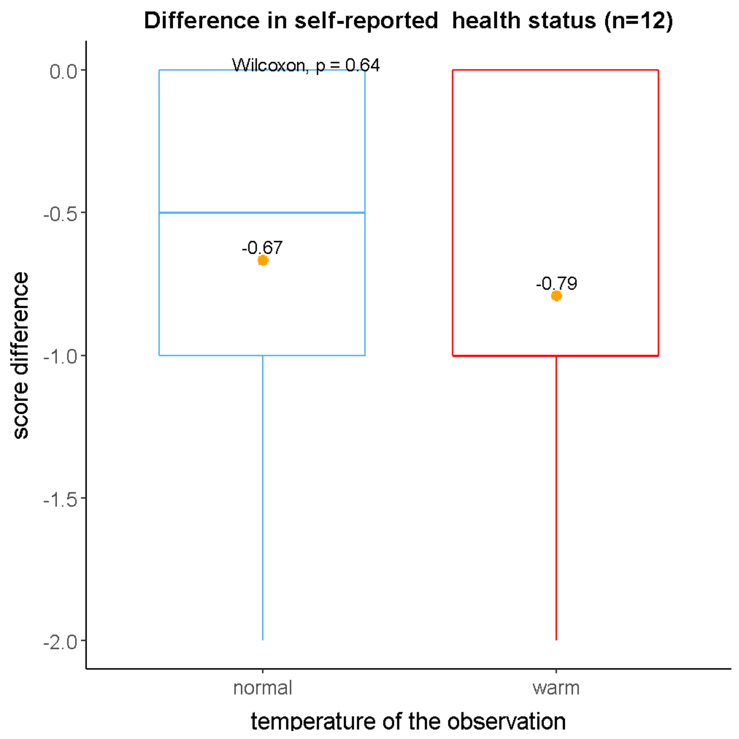

Figure S3. Box-whisker plots showing differences in self-reported health status, observed during the warm (summer; red) and normal (winter; blue) periods. Orange dots are the mean scores, and Wilcoxon-rank-sum-test was utilized for significance of differences.

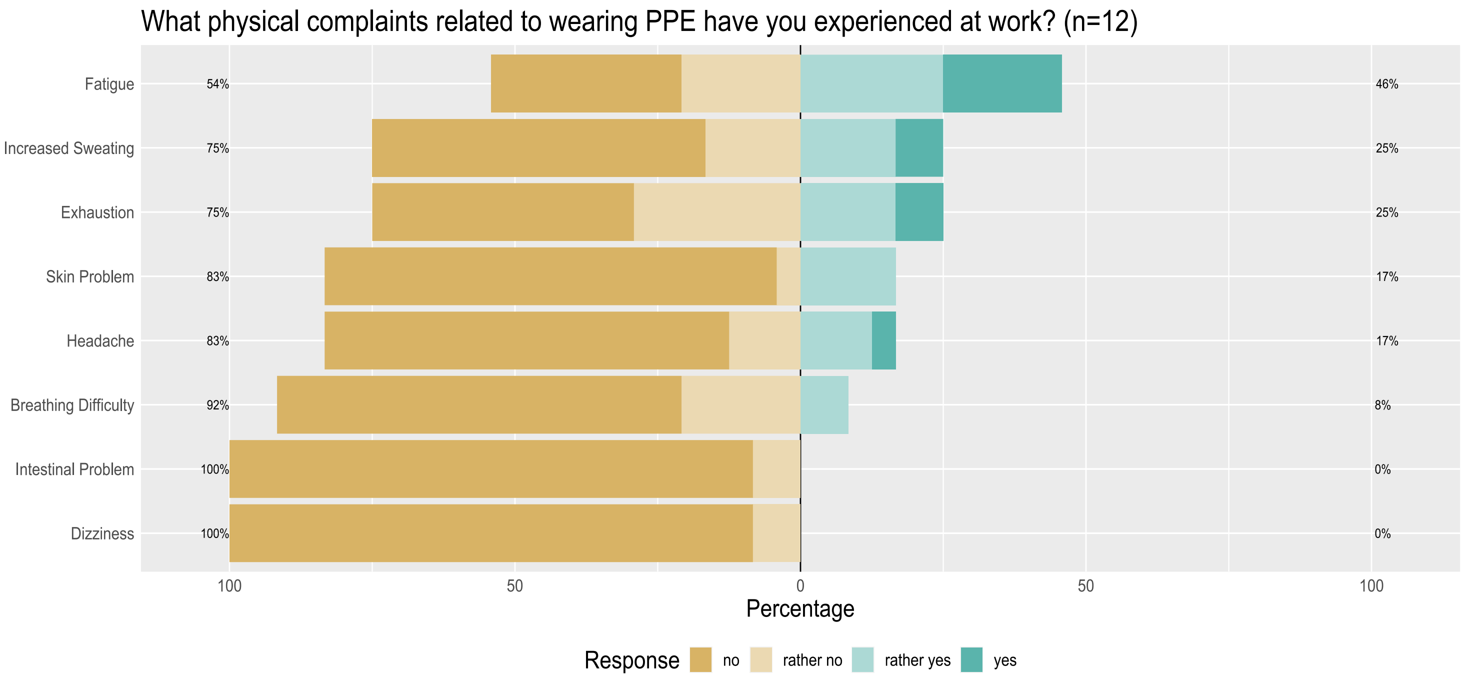

Figure S4. Likert scale distribution of perceived physical discomfort related to wearing PPE during the HCW work shift

Supplementary Table

Table S1. Perception of heat stress at the time of introductory session (*n* = 12)

**Heat stress in the workplace**	**n**
Heat stress at work	
Yes	10
Unfavorable temperature at work	
Daily	7
Weekly	3
Rarely	1
No answer	1
Unfavorable humidity at work	
Daily	8
Weekly	2
Rarely	1
No answer	1
Wearing at least one PPE at work*	
Yes	12
PPE-related impairment (open question)	
Sweating	5
Breathing difficulty	2
Dizziness	2
Skin problems	2
No problem noticed	5
Awareness of recommendation of health and safety at work on hot days?	
No	11
Own countermeasures (open question)	
Drinking more water	4
Taking breaks	4
Removing PPE	2
Not possible	1
No answer	4

*PPE = personal protective equipment
